# Changes in support for bans of illicit drugs, tobacco, and alcohol among adolescents and young adults in Europe, 2008–2014

**DOI:** 10.1007/s00038-017-1025-y

**Published:** 2017-08-09

**Authors:** Raffaele Palladino, Thomas Hone, Filippos T. Filippidis

**Affiliations:** Department of Primary Care and Public Health, Imperial College London, London, UK

**Keywords:** Adolescents, Drugs, Alcohol, Tobacco, Ban, Illicit

## Abstract

**Objectives:**

This study assessed the support for bans for tobacco, alcohol, and illicit drugs in adolescents and young adults across the European Union (EU).

**Methods:**

Data were analysed for the years 2008, 2011, and 2014 for 27 EU member states. 37,253 individuals aged 15–24 years were interviewed ascertaining their support for banning tobacco, alcohol, cannabis, cocaine, heroin, and ecstasy. Changes over time were assessed using multilevel logistic regression.

**Results:**

Support for banning heroin, ecstasy, and cocaine was constantly greater than 90%, although support fell over time. Support for cannabis ban declined (from 67.6% in 2008 to 53.7% in 2014) as well as support for alcohol ban (from 8.9% in 2008 to 6.9% in 2014) and tobacco ban (from 17.9% in 2008 to 16.5% in 2014).

**Conclusions:**

Support for banning substances among EU adolescents and young adults varied, with high support for heroin, cocaine, and ecstasy, but less support for banning cannabis, tobacco, and alcohol. There was reduction in support of banning all substances between 2008 and 2014, but this varied substantially between European countries.

**Electronic supplementary material:**

The online version of this article (doi:10.1007/s00038-017-1025-y) contains supplementary material, which is available to authorized users.

## Introduction

Globally, there is a drive to tackle tobacco, alcohol, and illicit drugs to reduce their harmful use. Consumption of these substances can have serious consequences for health and contribute to health inequities (Council of the European Union 2013; SAMHSA 2014). Public health policy regarding tobacco, alcohol, and illicit drugs depends strongly on populations’ attitudes to substance control (Andersen et al. 2007), and there is a need to understand how attitudes—especially of youth populations—have changed in recent years.

Illicit drugs, such as heroin, cocaine, ecstasy, and cannabis, are generally banned in European Union (EU) countries, and there are increasing steps to reduce consumption. The *EU Drugs Action Plan* (2013–2016) aims to reduce demand and supply through EU-wide and national coordination and international cooperation (Council of the European Union 2013). Even so, there are exceptions in certain member states—notably for cannabis—where regulation is more relaxed regarding consumption and possession (European Monitoring Centre for Drugs and Drug Addiction). In addition, the USA has recently relaxed legislation regarding cannabis with some states decriminalising or legalising usage (Pacula et al. 2015). The impact of recent changes in the legality of cannabis on attitudes to substance control is uncertain.

European countries have prioritised tackling tobacco and alcohol consumption in the World Health Organisation’s Regional Office for Europe’s *Health* 2020 *Strategy* (World health Organisation regional Office for Europe 2013). As important risk factors for chronic diseases, tobacco and alcohol are regulated, but there is wide variety in how this is implemented and enforced between EU member states. Alcohol remains an integral part of European culture with minimum drinking ages, taxes, and regulation varying between countries (World Health Organization regional Office for Europe 2012). Tobacco is heavily taxed and tobacco products display health warnings, and all EU member states have introduced forms of smoke-free legislation banning smoking in—at least some—public places (World Health Organization regional Office for Europe 2013). These strong efforts to tackle tobacco consumption have initiated discussion of the “tobacco endgame” and an eventual ban on tobacco in Europe (Warner 2013); however, strong public support would be required to pass such policies.

Adolescents and young adults (aged 15–24 years) are important for long-term strategies relating to drug, tobacco, and alcohol policies. Individuals are most likely to begin consumption at these ages and, for alcohol and tobacco, often form long-term habits. In most EU countries, the majority of smokers start regular smoking before the age of 18 (Filippidis et al. 2015; Goniewicz et al. 2016; Nadasan et al. 2016), with those in lower socio-economic groups more likely to smoke and more frequently exposed to the second-hand smoking (Lorant et al. 2017). In 2015, 21% of students 16 years old in Europe were smokers (ESPAD Group 2015), although a decreasing trend in smoking has been observed, particularly in Western Europe (Hublet et al. 2009). Furthermore, the estimated prevalence of heavy episodic drinking in Europe was 28.1% for those aged 12–16 years (Steketee et al. 2013) and likely to be even higher in older populations.

Whilst illicit drugs are markedly different to tobacco and alcohol both in legality and consumption patterns, adolescents in Europe present a vulnerable group with 18% of them reporting consumption of any illicit drug in their lifetime (ESPAD Group 2015), suggesting a lower perception of the risk (SAMHSA 2014). Single-country studies report even more alarming results (European Monitoring Centre for Drugs and Drug Addiction 2015). For example, a British study found that 6.6% of people aged 16–24 are frequent drug users—more than twice that for those aged 16–59 years (Lifestyles Statistics Team 2014).

Considering the key role played by adolescents and young adults in the European strategies to tackle tobacco, alcohol, and illicit drugs consumption, it is important to gauge their attitudes towards these substances, especially in the recent rapidly changing social and policy environment. This study uses three cross-sectional studies (2008, 2011, and 2014) to assess adolescents and young adults’ attitudes towards tobacco, alcohol, and drug bans in countries of the European Union.

## Methods

### Data source

We analysed data from three waves from the Flash Eurobarometer survey: wave 233 (*n* = 12,312; May 2008), wave 330 (*n* = 12,313; May 2011), and wave 401 (*n* = 12,628; June 2014) (Supplementary Table 1). These three waves were chosen as specifically focused on ‘young people and drugs’. Flash Eurobarometer surveys collect data from all member states of the EU through telephone interviews, using a three-stage random sampling method which is also used for standard Eurobarometer surveys (European Commission). In each household, the respondent was drawn at random following the ‘last birthday rule’. Eurobarometer does not publish response rates, but post-stratification and population weights are provided to account for the non-response rates and ensure that the samples are representative of the target populations (TNS Political and Social 2014). Each of these three waves consisted of individuals aged 15–24 years from 27 EU member states. Wave 401 also included respondents from Croatia, but they were excluded from our analysis, as Croatia was not part of the earlier waves.

### Measures

Outcome variables were defined by answering a specific question for each of the following substances: tobacco, alcohol, cannabis, cocaine, heroin, and ecstasy. Specifically, respondents were asked: “The sale of drugs such as cannabis, cocaine, ecstasy, and heroin is officially banned in all EU Member States. The sale of legal substances such as alcohol and tobacco is not prohibited but is regulated in all EU countries, which means for example that there is a minimum age limit for buying, limits in the concentration of active components, or licensed sales through specialised shops and pharmacies. Do you think the following substances should continue to be banned or should be banned, or should they be regulated?” Answers were: (a) should continue to be banned or should be banned; (b) should be regulated; (c) should be available without restrictions. Support for ban was coded as a binary variable.

Data were also collected on respondents’ age (15–17; 18–24 years), gender (male; female), area of residence (urban; rural), highest completed level of education (primary; secondary; higher), and current student status (yes; no).

### Statistical analysis

Proportions of respondents who supported bans of the assessed substances are presented as weighted percentages (%) with 95% Confidence Intervals (CI). Country-specific changes in the support for bans of the aforementioned substances over time were assessed with multivariate logistic regression models considering the survey year as main variable of interest (2008 as reference, 2011, 2014). Regression models were adjusted for respondents’ age, gender, education, current student status, and area of residence. In the pooled data analysis, to control for differences in country-specific legislation and prevention policies at the country level, a dummy variable for each country was included in the model. Sampling weights were employed to account for the complex, multi-stage design of the data set. Covariates used in adjusted analyses were tested for multicollinearity. The multicollinearity diagnostics (VIF) were all less than 5, indicating an assumption of reasonable independence among independent variables. Considering the high prevalence of the study outcomes, logistic regression results are presented as adjusted Prevalence Ratios (PR) with 95% CI. All analyses were performed with Stata 14.0.

## Results

More than nine out of ten adolescents and young adults responded that heroin, ecstasy, and cocaine should continue to be banned in all three waves. Support for bans on cannabis was notably lower, declining from 67.6% in 2008 to 53.7% in 2014. On the contrary, a small number of participants supported bans of alcohol (8.9, 7.0, and 6.9% in 2008, 2011, and 2014, respectively) and tobacco (17.9, 16.5, and 16.0% in 2008, 2011, and 2014, respectively) (Tables 1, 2). There was considerable variation between countries.

**Table 1:** Proportion of individuals aged 15–24 years who supported a ban of alcohol, tobacco, and cannabis in 27 European Union member states, 2008–2014

Country	Alcohol			Tobacco			Cannabis		
	2008	2011	2014	2008	2011	2014	2008	2011	2014
Austria	5.9 (4.2–8.4)	5.2 (3.5–7.6)	4.7 (3–7.5)	9.1 (6.9–12)	9.9 (7.5–12.9)	11.2 (8.4–14.7)	74 (69.8–77.7)	61.9 (57.5–66.1)	47 (41.9–52.1)
Belgium	4.6 (3.1–6.9)	7.8 (5.8–10.5)	8.8 (6.1–12.5)	17.8 (14.2–22.1)	17.5 (14.4–21.1)	19.7 (15.9–24.2)	63.1 (57.9–67.9)	48.3 (43.9–52.8)	61.2 (56.2–66)
Bulgaria	7.2 (5–10.3)	4.9 (3.3–7.1)	7.5 (5.5–10.1)	9.3 (6.8–12.7)	14.7 (11.9–18.1)	15.9 (12.9–19.4)	77.7 (73.6–81.3)	68.8 (64.6–72.7)	66.5 (62.1–70.6)
Cyprus (Republic)	8.9 (5.7–13.7)	9.1 (5.7–14)	4.3 (2.3–7.9)	20.2 (15.5–25.9)	21.6 (16.6–27.6)	12.4 (8.4–17.9)	83.8 (77.8–88.4)	82.7 (77–87.3)	72.6 (65.3–78.8)
Czech Republic	9.3 (7–12.3)	3.6 (2.3–5.6)	2.6 (1.4–4.6)	16 (12.9–19.5)	10.2 (7.7–13.2)	9.1 (6.6–12.3)	38.7 (34.4–43.2)	39.3 (35.1–43.8)	27.1 (23–31.7)
Denmark	1.2 (0.4–3.3)	2.6 (1.5–4.7)	1.6 (0.7–3.9)	11.4 (8.4–15.3)	13.8 (10.9–17.2)	10.3 (7.2–14.6)	70.3 (65.2–75)	64.2 (59.7–68.4)	57.9 (51.8–63.7)
Estonia	5.1 (3–8.6)	4.5 (2.4–8.3)	3 (1.8–4.7)	13.7 (9.6–19.2)	10.9 (7.4–15.8)	10.3 (7.9–13.5)	71.2 (64.8–76.9)	53.6 (47.1–59.9)	54.4 (49.7–59.1)
Finland	4.6 (3.1–6.9)	3.6 (1.9–6.4)	3.7 (2.3–6)	14 (11.1–17.6)	14.9 (11.7–18.8)	16.3 (13.3–19.9)	79.1 (74.9–82.7)	68 (63.1–72.5)	66 (61.5–70.2)
France	7.6 (5.5–10.6)	9.3 (7–12.2)	8.1 (6–10.9)	13.7 (10.7–17.3)	15 (12–18.5)	14.1 (11.2–17.6)	73 (68.4–77.1)	50.5 (45.9–55)	54.8 (50.3–59.2)
Germany	4.4 (2.9–6.6)	6.1 (4–9)	6.8 (4.4–10.5)	11.2 (8.6–14.4)	13.4 (10.5–17.1)	9.7 (6.9–13.4)	69.9 (65.4–74)	70.7 (66.2–74.9)	53.4 (48–58.7)
Greece	5.8 (4–8.3)	5 (3.4–7.3)	6.1 (4.2–8.7)	12.4 (9.7–15.7)	17.4 (14.3–21)	19 (15.6–23)	71.6 (67.5–75.4)	68.1 (63.7–72.1)	64.2 (59.6–68.5)
Hungary	7.4 (5.3–10.1)	6.7 (4.8–9.3)	6.3 (4.3–9.3)	16 (12.9–19.8)	15.2 (12.3–18.7)	15.4 (12.3–19.1)	76 (71.9–79.7)	72.4 (68.2–76.2)	69.2 (64.3–73.7)
Ireland	3.8 (2.4–5.8)	4.7 (3–7.2)	3.2 (1.9–5.2)	20.2 (16.9–24)	17.5 (14.2–21.4)	22.6 (19–26.5)	61.3 (56.8–65.5)	52.3 (47.5–57)	43.1 (38.7–47.6)
Italy	15.2 (12.2–18.9)	9.4 (7–12.4)	11 (8.5–14.1)	17.5 (14.3–21.3)	16.4 (13.2–20.2)	15.1 (12.1–18.6)	60.4 (55.8–64.8)	62.2 (57.4–66.8)	40.9 (36.4–45.5)
Latvia	13.8 (11–17.2)	5.5 (3.8–8)	8.2 (6.1–11)	21.9 (18.4–25.9)	12.3 (9.6–15.6)	17.6 (14.4–21.2)	76.1 (72–79.8)	64.2 (59.9–68.4)	72.1 (67.8–76)
Lithuania	10 (7.5–13.4)	9.4 (7.1–12.3)	17.1 (14–20.8)	15.3 (12.1–19)	21.1 (17.6–25)	24.6 (21–28.7)	80.2 (76.3–83.6)	74.8 (70.7–78.5)	71.1 (66.8–75)
Luxembourg	8.8 (5.7–13.5)	8.6 (5.4–13.5)	7.8 (4.7–12.7)	16 (11.6–21.5)	21.3 (16.4–27.2)	16.8 (12.1–22.9)	69.9 (63.3–75.8)	58.8 (52–65.2)	52.4 (45.2–59.5)
Malta	7.4 (4.4–12.2)	3.6 (1.9–6.7)	1.4 (0.5–3.9)	12 (8–17.8)	11.1 (7.7–15.6)	14.7 (9.7–21.5)	81.9 (76.2–86.5)	69.5 (63.3–75)	58.9 (51–66.4)
Poland	9.3 (6.9–12.3)	5 (3.4–7.3)	6 (4.1–8.7)	16.8 (13.6–20.5)	15.5 (12.5–19)	14.8 (11.7–18.4)	71 (66.6–75)	49.9 (45.4–54.3)	45.3 (40.9–49.9)
Portugal	12.1 (9.5–15.2)	5.2 (3.4–7.7)	8.2 (6–11.2)	19 (15.8–22.7)	15.4 (12.1–19.4)	18.8 (15.4–22.8)	68.9 (64.7–72.8)	52.6 (47.8–57.3)	65.9 (61.4–70.2)
Romania	22.3 (18.6–26.5)	14.9 (12–18.4)	15.1 (12.1–18.7)	31.8 (27.5–36.3)	30 (26–34.2)	28.9 (25–33.2)	92.2 (89.5–94.3)	87.9 (84.7–90.5)	85.8 (82.4–88.7)
Slovakia	14.9 (11.9–18.5)	6.4 (4.6–8.9)	10.4 (8–13.4)	21.9 (18.3–25.9)	11.1 (8.6–14.2)	15.1 (12.1–18.5)	68 (63.6–72.2)	53.3 (48.8–57.8)	46.2 (41.9–50.7)
Slovenia	6.5 (4.1–10.2)	7.8 (5–11.8)	3.1 (1.9–5.1)	8.1 (5.3–12.2)	13.4 (9.7–18.3)	8.7 (6–12.4)	63.3 (56.7–69.5)	49 (42.8–55.3)	36.0 (30.8–41.5)
Spain	13.7 (10.8–17.1)	9.3 (7–12.2)	6.9 (4.9–9.7)	25.1 (21.3–29.3)	28.9 (25.1–33.1)	23.7 (20–27.8)	59.2 (54.7–63.5)	57.5 (53–61.8)	52.8 (48.2–57.4)
Sweden	7.3 (5.3–10)	9.3 (6.1–13.8)	5.9 (4.1–8.4)	26.7 (22.9–30.8)	19 (14.4–24.6)	17.1 (13.8–21)	89.5 (86.3–92)	79.4 (74.1–83.9)	69.5 (64.7–73.9)
The Netherlands	1.3 (0.6–2.6)	3.2 (1.9–5.5)	0.8 (0.4–1.9)	8.5 (6.1–11.8)	7.1 (4.9–10.2)	8.6 (6–12.2)	45.9 (40.9–50.9)	32.9 (28.6–37.5)	47.2 (41.5–52.9)
United Kingdom	7.6 (5.5–10.5)	4 (2.4–6.6)	3.3 (2–5.5)	27.2 (23.2–31.6)	14.8 (11.6–18.6)	20 (16.6–23.9)	59.5 (54.9–63.9)	59.7 (54.8–64.3)	53.5 (48.9–58.1)
27 EU member states	8.9 (8.1–9.7)	7.0 (6.3–7.7)	6.9 (6.1–7.6)	17.9 (16.8–19.0)	16.5 (15.4–17.5)	16.0 (15.0–17.1)	67.6 (66.3–68.9)	60.2 (58.8–61.6)	53.7 (52.3–55.2)

Results are presented as weighted proportions (%) and 95% confidence intervals (CI)

**Table 2:** Proportion of individuals aged 15–24 years who supported a ban of cocaine, ecstasy, and heroin in 27 European Union member states, 2008–2014

Country	Cocaine			Ecstasy			Heroin		
	2008	2011	2014	2008	2011	2014	2008	2011	2014
Austria	97.2 (95.4–98.3)	94.2 (91.8–96)	94.5 (91.9–96.3)	96.1 (94–97.5)	94.6 (92.3–96.3)	94.6 (91.7–96.6)	97.2 (95.5–98.3)	95.9 (93.8–97.3)	96.6 (94.1–98.1)
Belgium	97.9 (95.9–99)	94.1 (91.6–95.9)	94.4 (91.8–96.3)	96.2 (93.7–97.7)	94.3 (91.8–96)	93.3 (90.5–95.4)	98.5 (96.4–99.4)	95.8 (93.5–97.3)	95.1 (92.6–96.8)
Bulgaria	95.8 (93.6–97.2)	95.8 (93.6–97.2)	95.2 (92.8–96.8)	92.4 (89.6–94.5)	93.4 (90.9–95.3)	93.1 (90.5–95)	95.6 (93.3–97.1)	97.5 (95.7–98.5)	96.7 (94.6–98)
Cyprus (Republic)	92.6 (87.9–95.6)	94.3 (90–96.9)	94.9 (90.7–97.2)	90.3 (85.4–93.7)	90.8 (86.3–94)	94.7 (90.1–97.2)	94 (89.6–96.7)	95.8 (91.8–97.9)	96.3 (92.4–98.2)
Czech Republic	93.1 (90.3–95.1)	95.9 (93.7–97.4)	93.3 (90.6–95.3)	81.2 (77.4–84.5)	86.9 (83.5–89.6)	85.9 (82.2–88.8)	94.8 (92.3–96.5)	97.4 (95.5–98.5)	95.6 (93.1–97.2)
Denmark	96.9 (94.2–98.4)	94.6 (92.2–96.3)	90.8 (86.5–93.8)	98 (96–99)	94.8 (92.4–96.5)	93.3 (89.9–95.7)	97.9 (95.4–99.1)	94.7 (92.3–96.4)	93.5 (90–95.9)
Estonia	97.5 (94.1–99)	96.5 (93.4–98.2)	95.4 (92.9–97.1)	94.3 (90.3–96.7)	93.1 (88.9–95.8)	94.6 (92.1–96.3)	98.2 (94.6–99.4)	97.4 (94.6–98.8)	97 (95–98.3)
Finland	96.9 (94.9–98.2)	96.8 (94.2–98.2)	95.8 (93.6–97.3)	95.6 (93.3–97.2)	96.8 (94.5–98.1)	92.7 (90–94.7)	97.7 (95.8–98.7)	98.4 (96.5–99.3)	97.7 (95.9–98.8)
France	97.1 (95.1–98.3)	93.8 (91.3–95.7)	94.3 (91.8–96.1)	98.2 (96.2–99.1)	91.6 (88.7–93.8)	93.7 (91.2–95.5)	98.7 (97.1–99.4)	96.4 (94.3–97.7)	96.8 (94.8–98)
Germany	96.7 (94.8–98)	96.3 (94.1–97.7)	93.3 (90.2–95.5)	97.5 (95.6–98.6)	96.2 (94.1–97.6)	93.9 (90.7–96.1)	97.5 (95.6–98.6)	97.6 (95.6–98.7)	96.1 (94–97.5)
Greece	94.8 (92.5–96.4)	95 (92.6–96.6)	91.1 (88.1–93.3)	90.2 (87.3–92.6)	91.3 (88.5–93.5)	87 (83.3–89.9)	96.5 (94.4–97.8)	97.4 (95.5–98.4)	95.3 (93.1–96.9)
Hungary	98.1 (96.4–99)	96.4 (94.3–97.7)	96.1 (93.2–97.7)	94 (91.3–95.9)	92.6 (89.9–94.6)	92.5 (89.3–94.8)	98.2 (96.4–99.2)	96.7 (94.7–97.9)	96.7 (94.2–98.2)
Ireland	94.8 (92.4–96.4)	93.6 (90.9–95.5)	91.2 (88.3–93.5)	94.5 (92.1–96.1)	92.7 (90–94.7)	89.4 (86.3–91.9)	96.7 (94.7–97.9)	96.9 (94.8–98.1)	93.2 (90.6–95.1)
Italy	97.4 (95.5–98.6)	93.9 (91–95.9)	93.6 (90.9–95.6)	97.4 (95.4–98.5)	94.1 (91.2–96.1)	94.5 (92–96.3)	97.8 (95.9–98.8)	95.1 (92.4–96.8)	95.5 (93.1–97.1)
Latvia	97.7 (95.9–98.7)	95.1 (92.7–96.7)	94.8 (92.4–96.5)	92.5 (89.8–94.5)	90.9 (88–93.2)	94.8 (92.4–96.5)	98.6 (97.2–99.3)	96.9 (94.9–98.1)	96.8 (94.7–98.1)
Lithuania	98.5 (97–99.3)	99.3 (98–99.7)	96.5 (94.4–97.8)	97.5 (95.8–98.6)	98.4 (96.8–99.2)	94.8 (92.5–96.5)	98.7 (97.1–99.4)	99.1 (97.9–99.6)	97.4 (95.6–98.5)
Luxembourg	96.2 (92.9–97.9)	95.8 (92.6–97.7)	95.1 (91.1–97.4)	95 (91.5–97)	94.2 (90.5–96.5)	93.9 (89.2–96.6)	96.8 (93.7–98.4)	97.6 (94.6–99)	96.5 (92.7–98.3)
Malta	92.5 (87.8–95.5)	93.5 (89.7–96)	90.8 (85.1–94.5)	92.4 (87.7–95.4)	92.4 (88.4–95.1)	92.1 (86.9–95.3)	93.7 (89.3–96.3)	94.2 (90.7–96.5)	94.1 (89.8–96.7)
Poland	96 (93.5–97.5)	93.2 (90.6–95.1)	90.7 (87.7–93.1)	95.9 (93.6–97.4)	90.5 (87.6–92.8)	84.5 (80.9–87.6)	96.8 (94.4–98.2)	97.1 (95.3–98.3)	93.3 (90.5–95.3)
Portugal	94.5 (92.1–96.2)	91.5 (88.5–93.8)	91.7 (88.7–93.9)	90.9 (88–93.1)	86.9 (83.3–89.8)	91.2 (88.3–93.5)	95.6 (93.3–97.1)	92.3 (89.4–94.5)	93.6 (90.9–95.5)
Romania	98.4 (97–99.2)	97.5 (95.7–98.6)	95.4 (93.2–96.9)	95.4 (93–97.1)	93.9 (91.5–95.7)	93.9 (91.5–95.7)	99.5 (98.5–99.8)	98.6 (97.1–99.3)	97.5 (95.7–98.5)
Slovakia	97.6 (95.7–98.7)	98.1 (96.4–99)	94.8 (92.5–96.4)	93.6 (90.9–95.5)	92.3 (89.5–94.3)	90.1 (87.1–92.4)	98.3 (96.6–99.2)	98.1 (96.4–99)	96.3 (94.3–97.7)
Slovenia	93.7 (89.4–96.4)	91 (86.6–94)	83.4 (78.9–87.1)	89.3 (84.2–92.9)	89.8 (85.2–93.1)	84.4 (80.1–87.9)	95.9 (91.8–98)	94.2 (90.4–96.6)	87.9 (83.9–91.1)
Spain	93.6 (91.2–95.5)	94.2 (91.7–95.9)	94.2 (91.7–96)	93.6 (91.1–95.5)	93.7 (91.1–95.5)	94.3 (91.8–96.1)	95.5 (93.3–97)	95.7 (93.5–97.1)	96.3 (94.1–97.7)
Sweden	98.1 (96.3–99)	96.2 (92.5–98.1)	95.4 (92.6–97.1)	97.5 (95.8–98.5)	95.9 (92.2–97.9)	94.8 (91.8–96.7)	98.1 (96.3–99)	97 (93.1–98.8)	96.4 (93.9–97.9)
The Netherlands	88.7 (84.8–91.7)	86 (82.3–89)	86.6 (82.1–90)	84.2 (79.9–87.7)	80.3 (76.2–83.9)	85.7 (81.7–88.9)	91.7 (87.9–94.3)	88.6 (85.2–91.4)	92.7 (89.2–95.1)
United Kingdom	91.8 (89–93.9)	92.2 (89.2–94.4)	91.9 (89–94.1)	90.7 (87.7–93.1)	91.3 (88.1–93.7)	89 (85.7–91.6)	95.9 (93.7–97.3)	96.5 (94.3–97.8)	95.6 (93.2–97.2)
27 EU member states	95.6 (95.0–96.2)	94.2 (93.5–94.8)	93.1 (92.3–93.8)	94.7 (94.1–95.2)	92.5 (91.8–93.3)	91.7 (90.9–92.5)	97.1 (96.6–97.5)	96.3 (95.8–96.8)	95.7 (95.1–96.2)

Results are presented as weighted proportions (%) and 95% confidence intervals (CI)

**Fig. 1:**
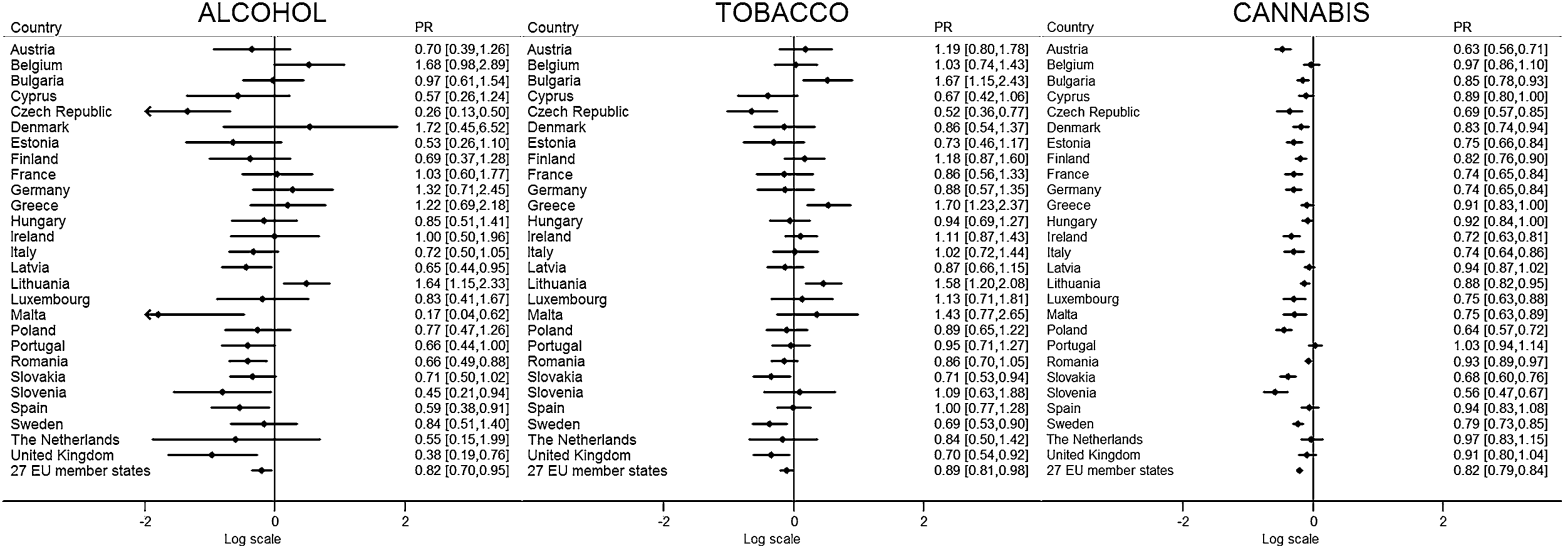
Change in support for bans of alcohol, tobacco, and cannabis in 27 European Union member states between 2008 and 2014. *Notes* Results from multivariate logistic regression models. Results are presented as adjusted prevalence ratios (PR) and 95% confidence intervals (CI) in log scale. The *right column* reports results without the log transformation

**Fig. 2:**
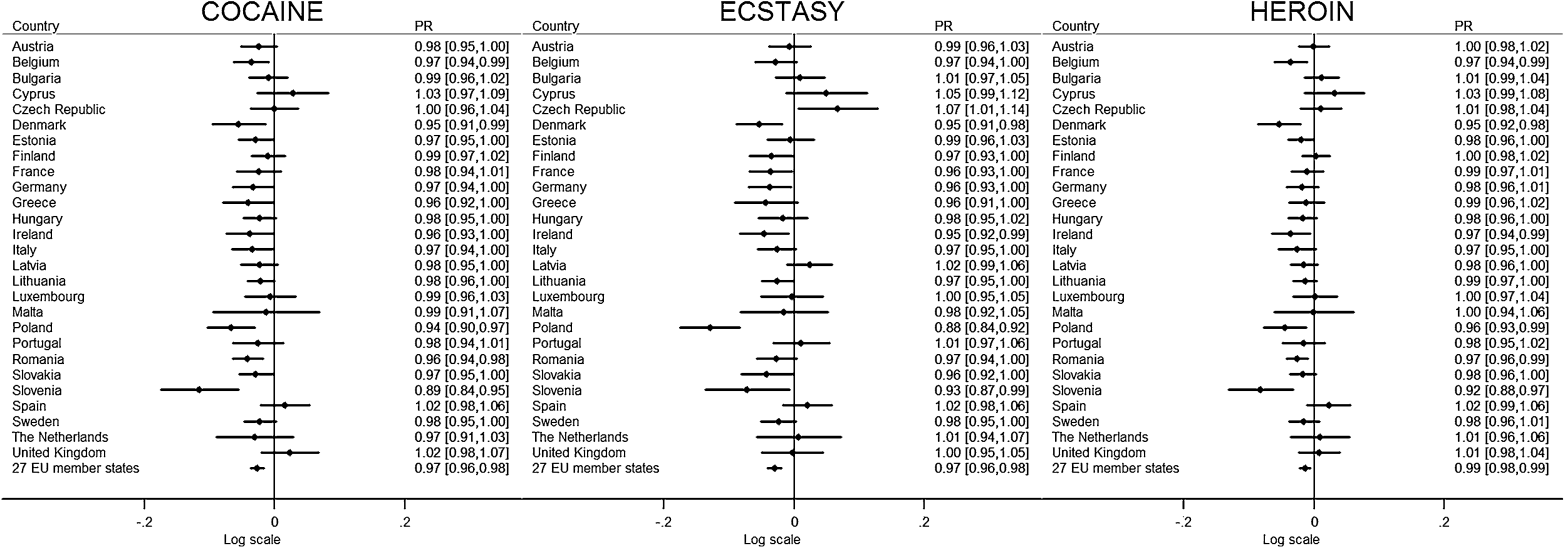
Change in support for bans of cocaine, ecstasy, and heroin in 27 European Union member states between 2008 and 2014. *Notes* Results from multivariate logistic regression models. Results are presented as adjusted prevalence ratios (PR) and 95% confidence intervals (CI) in log scale. The *right column* reports results without the log transformation

After adjusting for age, gender, area of residence, education, and current student status, in adolescents and young adults in the EU, compared to 2008, the support for cannabis bans was 10% lower in 2011 and 18% lower in 2014 (PR 0.90, 95% CI 0.87–0.93 in 2011 and PR 0.82, 95% CI 0.79–0.84 in 2014), and the support for cocaine bans was 2% lower in 2011 and 3% lower in 2014 (PR 0.98, 95% CI 0.98–0.99 in 2011 and PR 0.97, 95% CI 0.98–00.99 in 2014). The support for heroin bans was 1% lower in both 2011 and 2014 (PR 0.99, 95% CI 0.99–1.00 in 2011 and PR 0.99, 95% CI 0.98–0.99 in 2014), and the support for ecstasy bans was 2% lower in 2011 and 3% lower in 2014 (PR 0.98, 95% CI 0.97–0.99 in 2011 and PR 0.97, 95% CI 0.96–0.98 in 2014) (Figs. 1, 2; Supplementary Table 2). Support for alcohol bans was also 8% lower in both 2011 (PR 0.82, 95% CI 0.71–0.94) and 2014 (PR 0.82, 95% CI 0.70–0.85) in comparison to 2008. Compared to 2008, there was no difference in 2011 to support for tobacco bans, but support was 11% lower in 2014 (PR 0.89, 95% CI 0.81–0.98).

In general, females were more supportive of bans for all substances, compared to men (PR ranging from 1.01 for heroin to 1.48 for alcohol), while adults (over 18 years of age) gave lower support for the ban of tobacco, and cannabis compared to adolescents. The proportion of respondents living in rural areas supporting bans for all substances except for alcohol was higher than those living in cities. Finally, current students and those who had completed higher education were generally less supportive of bans than those who were not studying at the time of the surveys and those who had only completed primary education. However, there were some exceptions, such as tobacco and cocaine, in which there was no significant difference between respondents of different educational levels (Supplementary Table 1).

By individual countries, there were notable trends between 2008 and 2014. There were statistically significant declines in support for banning cannabis, cocaine, heroin, and ecstasy for 20, 12, 7, and 9 countries, respectively (Figs. 1, 2). Support for banning all four illicit substances did not fall in seven countries—The Netherlands, United Kingdom, Spain, Portugal, Cyprus, Hungary, and Latvia. For Denmark, Ireland, Poland, and Slovenia, there were significant falls in support for bans for all four illicit substances (cocaine, heroin, ecstasy, and cannabis). Of these, Slovenia had the largest falls in supports of substance bans, resulting in the lowest support for bans of cannabis, cocaine, and heroin among the 27 countries in 2014. Between 2008 and 2014, support for substance bans in Slovenia declined 44% for cannabis (from 63.3 to 36.0%; PR 0.56, 95% CI 0.47–0.67), 11% cocaine (from 93.7 to 83.4%; PR 0.89, 95% CI 0.84–0.95), and 8% heroin (from 95.5 to 87.9%; PR 0.92, 95% CI 0.88–0.97)—which were the largest falls in the 27 countries—and 7% for ecstasy (from 89.3 to 84.4%; PR 0.93, 95% CI 0.87–0.99)—the second largest decline. Likewise, in Poland, there were large declines in support for all four substances and lower overall support compared to other countries, whilst both Denmark and Ireland had lower overall support notably for cocaine and heroin. Nonetheless, there was still high support (with most countries over 90%) for bans across countries for cocaine, heroin, and ecstasy.

Cannabis showed the greatest reductions in support for bans—20 countries showing reductions—with over 20% falls in support (from 2008 to 2014) for Austria, Malta, Poland, Slovenia, Slovakia, and Sweden. The lowest support in 2014 for banning cannabis was in the Czech Republic (27.1, 38.7% in 2008), Slovenia (36.0, 63.3% in 2008), Italy (40.9, 60.4% in 2008), and Ireland (43.1, 61.3% in 2008), whilst the highest was in Romania (85.8, 92.2% in 2008), Cyprus (72.6, 83.8% in 2008), Latvia (72.1, 76.1% in 2008), and Lithuania (71.1, 80.2% in 2008).

There were less significant changes in support for bans for tobacco and alcohol which remained generally low across the 27 countries. In the United Kingdom, Czech Republic, and Slovakia, there was decreased support for bans of both alcohol and tobacco, whilst in Spain, Malta, Slovenia, and Romania, there were declines for alcohol, and in Sweden for tobacco. For tobacco, the countries experiencing declines had higher (>20%) than average support for tobacco bans in 2008. For alcohol, many countries with higher support (>15%) for bans in 2008 experienced declines in support, with support in Romania declining 34–15.1% in 2014 (22.3% in 2008; PR 0.66, 95% CI 0.49–0.88).

In very few countries, there was an increase in for support in banning substances. In the Czech Republic, support for banning ecstasy increased by 7% (from 81.2% in 2008—the lowest of the 27 countries—to 85.9% in 2014; PR 1.07, 95% CI 1.01–1.14). In Lithuania, there was 64% increase in support for an alcohol ban (from 10.0 to 17.1%; PR 1.64, 95% CI 1.15–2.33) making it the country with the highest support for a ban in 2014. In three countries—Greece, Lithuania, and Bulgaria—there were statistically significant increases in support of a tobacco ban from 12.4% in 2008 to 19.0% in 2014, 15.3–24.6%, and 9.3–15.9%, respectively.

## Discussion

The majority of adolescents and young adults (over 90%) in the EU support bans of heroin, cocaine, and ecstasy, and over half support cannabis ban. Conversely, support for tobacco and alcohol bans is low. Since 2008, support for bans of all substances has fallen although only marginally for tobacco. Even though there is variability between European countries regarding support for bans and the changes that have occurred over time, there is still strong consensus on support for banning illicit drugs, but a little support for alcohol and tobacco bans.

The strong support for banning “hard” drugs—heroin, cocaine, and ecstasy – infers that the risks associated with these are well established among European adolescents and young adults. The lower, yet still substantial, support for a cannabis ban may be due to the perception of cannabis as a less risky drug. This finding is in line with the results from recent studies in USA which reported a decreasing trend in perceived risk associated with cannabis consumption (Okaneku et al. 2015), especially in adolescents (National Institute on Drug Abuse 2016). At the individual country level, there were large declines for support of banning cannabis in most countries, although there is not clear relationship with the legality (or de-criminalised nature) of cannabis possession (European Monitoring Centre for Drugs and Drug Addiction). It is also likely that the relaxed regulation of cannabis in some European countries, i.e., Denmark and The Netherlands, explains the lower support for a ban. The increase in medicinal cannabis use has been shown to have no impact on cannabis use by youth populations (Hasin et al. 2015); however, it may have had a small influence in changing attitudes towards cannabis.

This study shows low support for a tobacco ban among European adolescents and young adults, with support for this ban decreasing over time. This is in contrast to the previous findings from international studies reporting increasing support for tobacco regulation and tobacco-free policies among young adults (Jaine et al. 2015; Ling et al. 2007, 2009; National Institute on Drug Abuse 2016; Waller et al. 2004), in line with overall decreases in smoking prevalence worldwide. Examining trends by countries between 2008 and 2014—some countries (Czech Republic, Slovakia, Sweden, and the United Kingdom) showed significantly lower support for bans, whilst other countries (Bulgaria, Greece, and Lithuania) showed increased support. This may in part be explained by the introduction of smoke-free legislation in Greece (2010) and Bulgaria (2012)—during the study period, whilst in the UK and Sweden, smoke-free legislation was introduced before 2008, and has yet to be comprehensibly introduced in Czech Republic and Slovakia. These unclear results may also be due to varying sentiments toward smoke-free legislation and banning a substance. Adolescents and young adults may indeed support smoke-free policies and greater restrictions, but are not supportive of policies that would prohibit personal consumption of tobacco. Support was only assessed with a single question in this survey, whereas more elaborate approaches have been used before to comprehensively assess support for tobacco control measures (Schumann et al. 2006; Velicer et al. 1994). A more detailed questionnaire would allow further exploration of attitudes and should be addressed in future studies, not only with regard to tobacco bans, but also a wider range of policies for tobacco and other substances.

In a broader context, the variability in support of bans between EU member states highlights the differences in attitudes within the EU. Quite often, policies related to the regulation of substances in the European Union are being decided at a central level, for example with the recent revision of the Tobacco Products Directive (The European Parliament and the Council of the European Union 2014). While this allows the dissemination of good practices and harmonizes legislation throughout the EU, some policies may not be equally relevant or effective in all member states. Cultural factors and the local population’s support may play a crucial role in effectively enforcing legislation; hence, these should be taken into account when designing policy interventions in specific countries.

This study also demonstrates low and decreasing support over time for an alcohol ban, in line with the previous European studies (Steketee et al. 2013). This is likely to reflect that the position alcohol has as a deeply rooted habit in European culture, especially in North East Europe, with high consumption even at young age (Holubcikova et al. 2017). The only country with increased support for an alcohol ban was Lithuania, which may be explained by its alcohol consumption and alcohol-related deaths as amongst the highest globally.

The prevalence of support for banning these substances was higher among females and those living in rural areas. These differences may be explained by findings from previous studies showing the perceived risk associated with substances misuse is lower in males (Okaneku et al. 2015) and that those living in urban areas are more exposed to possible substances abuse (especially alcohol) due to, among other factors, increased substances abuse in older population in these settings (Chuang et al. 2009; Lifestyles Statistics Team 2014).

### Strengths and limitations

We used a robust survey that collects individuals’ attitudes and characteristics over time from representative European populations. While different samples were collected in each survey, they were representative of the population aged 15–24 years of age. Considering that the composition of this age group is unlikely to have changed within such a short period of time, and we consider comparisons between waves as meaningful. A limitation of this study is the use of self-reported data that can introduce bias from recall or unwillingness to share certain information. In particular, misreporting may be higher for those of lower socio-economic and education backgrounds (Palladino et al. 2016). Moreover, telephone-based interviews may also introduce selection bias associated with potential coverage. Our study population covered a wide age range including both adolescents and young adults, with different exposures to illegal substances and subsequent attitudes towards bans (Cheng et al. 2016). In addition, large surveys such as the Global Youth Tobacco Survey (Center for Disease Control and Prevention) did not specifically examine the adolescents’ attitude towards banning tobacco products as they focused on outdoor/indoor smoking ban; therefore, a comparison was not possible. Finally, we have no information of individuals’ usage patterns of alcohol, drugs, and tobacco. It is likely that users would be less supportive of any ban, and such information would provide further understanding of the trends. Future studies could explore whether the trends that we have detected in our analysis are mediated by changes in substance use.

### Policy implications

Whilst support remains high for banning most illicit substances, the fall in support over time in adolescents and young adults poses challenges for policy makers, considering that policies to enforce bans may be dependent on public support (Andersen et al. 2007). There is need to fully communicate the risks of illicit substance misuse to adolescents and young adults. In addition, ensuring adherence to new legislation restricting substances such as tobacco and alcohol may be problematic with such little support. Tackling alcohol and tobacco consumption is a clear priority in Europe and globally, and policy makers must explore a wide range of interventions not involving bans to control consumption.

## Electronic supplementary material

Below is the link to the electronic supplementary material.

Supplementary material 1 (DOCX 17 kb)

## References

[CR1] Andersen PA, Buller DB, Voeks JH, Borland R, Helme D, Bettinghaus EP (2007). Predictors of government officials’ support for youth tobacco control policies. J Public Health Manag Pract.

[CR2] Center for Disease Control and Prevention Global Youth Tobacco Survey (GYTS). https://nccd.cdc.gov/GTSSDataSurveyResources/Ancillary/Documentation.aspx?SUID=1&DOCT=1. Accessed 13 Jan 2017

[CR3] Cheng HG, Cantave MD, Anthony JC (2016). Alcohol experiences viewed mutoscopically: newly incident drinking of twelve- to twenty-five-year-olds in the United States, 2002–2013. J Stud Alcohol Drugs.

[CR4] Chuang YC, Ennett ST, Bauman KE, Foshee VA (2009). Relationships of adolescents’ perceptions of parental and peer behaviors with cigarette and alcohol use in different neighborhood contexts. J Youth Adolesc.

[CR5] Council of the European Union (2013) EU Action Plan on Drugs 2013–2016 vol OJ C 351 of 30.11.2013

[CR6] ESPAD Group (2015). ESPAD Report 2015—results from the European School Survey Project on alcohol and other drugs.

[CR7] European Commission Eurobarometer surveys—last updates for each type of survey. http://ec.europa.eu/public_opinion/index_en.htm. Accessed 10 Feb 2016

[CR8] European Monitoring Centre for Drugs and Drug Addiction (2015) European drug report—trends and developments. In: Publications Office of the European Union

[CR9] European Monitoring Centre for Drugs and Drug Addiction Legal topic overviews: possession of cannabis for personal use. In. http://www.emcdda.europa.eu/legal-topic-overviews/cannabis-possession-for-personal-use#countries. Accessed 03 Feb 2016

[CR10] Filippidis FT, Agaku IT, Vardavas CI (2015). The association between peer, parental influence and tobacco product features and earlier age of onset of regular smoking among adults in 27 European countries. Eur J Public Health.

[CR11] Goniewicz ML (2016). Dual use of electronic and tobacco cigarettes among adolescents: a cross-sectional study in Poland. Int J Public Health.

[CR12] Hasin DS (2015). Medical marijuana laws and adolescent marijuana use in the USA from 1991 to 2014: results from annual, repeated cross-sectional surveys. Lancet Psychiatry.

[CR13] Holubcikova J, Kolarcik P, Madarasova Geckova A, Joppova E, van Dijk JP, Reijneveld SA (2017). Young adolescents who combine alcohol and energy drinks have a higher risk of reporting negative behavioural outcomes. Int J Public Health.

[CR14] Hublet A (2009). Association between tobacco control policies and smoking behaviour among adolescents in 29 European countries. Addiction.

[CR15] Jaine R, Healey B, Edwards R, Hoek J (2015). How adolescents view the tobacco endgame and tobacco control measures: trends and associations in support among 14–15 year olds. Tob Control.

[CR16] Lifestyles Statistics Team (2014) Statistics on drug misuse, England 2014. http://content.digital.nhs.uk/catalogue/PUB15943. Accessed 03 Feb 2016

[CR17] Ling PM, Neilands TB, Glantz SA (2007). The effect of support for action against the tobacco industry on smoking among young adults. Am J Public Health.

[CR18] Ling PM, Neilands TB, Glantz SA (2009). Young adult smoking behavior: a national survey. Am J Prev Med.

[CR19] Lorant V (2017). Social network and inequalities in smoking amongst school-aged adolescents in six European countries. Int J Public Health.

[CR20] Nadasan V (2016). Use of electronic cigarettes and alternative tobacco products among Romanian adolescents. Int J Public Health.

[CR21] National Institute on Drug Abuse (2016) Drug use trends remain stable or decline among teens. https://www.drugabuse.gov/news-events/news-releases/2015/12/drug-use-trends-remain-stable-or-decline-among-teens. Accessed 31 May 2016

[CR22] Okaneku J, Vearrier D, McKeever RG, LaSala GS, Greenberg MI (2015). Change in perceived risk associated with marijuana use in the United States from 2002 to 2012. Clin Toxicol (Phila).

[CR23] Pacula R, Jacobson M, Maksabedian EJ (2015). In the weeds: a baseline view of cannabis use among legalizing states and their neighbours. Addiction.

[CR24] Palladino R, Tayu Lee J, Ashworth M, Triassi M, Millett C (2016). Associations between multimorbidity, healthcare utilisation and health status: evidence from 16 European countries. Age Ageing.

[CR25] SAMHSA (2014) Results from the 2013 National Survey on Drug Use and Health: Summary of National Findings

[CR26] Schumann A, John U, Thyrian JR, Ulbricht S, Hapke U, Meyer C (2006). Attitudes towards smoking policies and tobacco control measures in relation to smoking status and smoking behaviour. Eur J Public Health.

[CR27] Steketee M, Jonkman H, Berten H, Vettenburg N (2013) Alcohol use among adolescents in Europe. Utrecht

[CR28] The European Parliament and the Council of the European Union (2014) Directive of the European Parliament and of the Council on the approximation of the laws, regulations and administrative provisions of the Member States concerning the manufacture, presentation and sale of tobacco and related products and repealing. vol Directive 2001/37/EC

[CR29] TNS Political & Social (2014). Flash eurobarometer 401—young people and drugs.

[CR30] Velicer WF, Laforge RG, Levesque DA, Fava JL (1994). The development and initial validation of the smoking policy inventory. Tob Control.

[CR31] Waller BJ, Cohen JE, Ashley MJ (2004). Youth attitudes towards tobacco control: a preliminary assessment. Chronic Dis Can.

[CR32] Warner KE (2013). An endgame for tobacco?. Tobacco Control.

[CR33] World health Organisation regional Office for Europe (2013) Health 2020. A European policy framework and strategy for the 21st century. WHO Regional Office for Europe Copenhagen, Denmark

[CR34] World Health Organization regional Office for Europe (2012). European action plan to reduce the harmful use of alcohol 2012–2020.

[CR35] World Health Organization regional Office for Europe (2013). European tobacco control status report 2013.

