# Supplementary material for: Changes in support for bans of illicit drugs, tobacco, and alcohol among adolescents and young adults in Europe, 2008–2014
Source: Int J Public Health. 2017 Aug 9;63(1):23–31. doi: 10.1007/s00038-017-1025-y (PMC5766717; doi:10.1007/s00038-017-1025-y)
Supplement: Supplementary file 1 — Supplementary material 1 (DOCX 17 kb) [file 38_2017_1025_MOESM1_ESM.docx]

**International Journal of Public Health**

**Changes in support for bans of illicit drugs, tobacco and alcohol among adolescents and young adults in Europe, 2008-2014**

Supplementary Material

Supplementary Table 1. Sample sizes of the 27 European Union member states in the Flash Eurobarometer survey. Data are analysed from wave 233 (2008), wave 330 (2011), and wave 401 (2014).

| **COUNTRY** | **Sample size** | | |
| --- | --- | --- | --- |
|  | **2008** | **2011** | **2014** |
| **Austria** | 500 | 501 | 501 |
| **Belgium** | 500 | 502 | 500 |
| **Bulgaria** | 502 | 508 | 500 |
| **Cyprus (Republic)** | 252 | 250 | 202 |
| **Czech Republic** | 505 | 503 | 500 |
| **Denmark** | 503 | 509 | 500 |
| **Estonia** | 250 | 253 | 500 |
| **Finland** | 502 | 502 | 500 |
| **France** | 504 | 501 | 500 |
| **Germany** | 517 | 503 | 500 |
| **Greece** | 500 | 504 | 500 |
| **Hungary** | 500 | 500 | 500 |
| **Ireland** | 500 | 500 | 500 |
| **Italy** | 500 | 501 | 501 |
| **Latvia** | 504 | 501 | 505 |
| **Lithuania** | 501 | 501 | 500 |
| **Luxembourg** | 250 | 250 | 200 |
| **Malta** | 250 | 257 | 201 |
| **Poland** | 501 | 503 | 510 |
| **Portugal** | 507 | 502 | 503 |
| **Romania** | 505 | 500 | 501 |
| **Slovakia** | 501 | 502 | 500 |
| **Slovenia** | 251 | 253 | 501 |
| **Spain** | 500 | 503 | 502 |
| **Sweden** | 504 | 502 | 500 |
| **The Netherlands** | 503 | 502 | 500 |
| **United Kingdom** | 500 | 500 | 501 |
| **27 EU member states** | 12,312 | 12,313 | 12,628 |

Supplementary Table 2. Factors associated with support for bans of illicit drugs, tobacco and alcohol in 27 European Union member states, 2008-2014.

|  |  | **Tobacco**  **PR (95% CI)** | **Alcohol**  **PR (95% CI)** | **Cannabis**  **PR (95% CI)** | **Cocaine**  **PR (95% CI)** | **Heroin**  **PR (95% CI)** | **Ecstasy**  **PR (95% CI)** |
| --- | --- | --- | --- | --- | --- | --- | --- |
| Survey year | | | | | | | |
|  | 2008 (referent) |  |  |  |  |  |  |
|  | 2011 | 0.92 (0.84 – 1.00) | **0.82 (0.71 - 0.94)** | **0.90 (0.87 - 0.93)** | **0.98 (0.98 - 0.99)** | **0.99 (0.99 – 1.00)** | **0.98 (0.97 - 0.99)** |
|  | 2014 | **0.89 (0.81 – 0.98)** | **0.82 (0.70 - 0.85)** | **0.82 (0.79 - 0.84)** | **0.97 (0.96 - 0.98)** | **0.99 (0.98 - 0.99)** | **0.97 (0.96 - 0.98)** |
| **Age (in years)** | | | | | | | |
|  | 15-17(referent) |  |  |  |  |  |  |
|  | 18-24 | **0.79 (0.71 - 0.88)** | 0.92 (0.77 – 1.10) | **0.86 (0.83 - 0.90)** | 1.00 (0.98 - 1.01) | 1.00 (0.99 - 1.01) | 1.00 (0.98 - 1.01) |
| **Gender** | | | | | | | |
|  | Male (referent) |  |  |  |  |  |  |
|  | Female | 1.04 (0.97 - 1.12) | **1.48 (1.32 - 1.67)** | **1.12 (1.09 - 1.15)** | **1.02 (1.01 - 1.03)** | **1.01 (1.01 - 1.02)** | **1.03 (1.03 - 1.03)** |
| **Area of residence** | | | | | | | |
|  | Urban (referent) |  |  |  |  |  |  |
|  | Rural | **1.10 (1.02 - 1.20)** | 1.05 (0.93 - 1.19) | **1.12 (1.09 - 1.15)** | **1.01 (1.00 - 1.02)** | **1.01 (1.01 - 1.02)** | **1.01 (1.00 - 1.02)** |
| Level of education | | | | | | | |
|  | Primary (referent) |  |  |  |  |  |  |
|  | Secondary | 0.99 (0.90 - 1.11) | 0.91 (0.76 – 1.08) | 1.00 (0.96 – 1.03) | 1.00 (0.99 - 1.01) | 1.00 (0.99 - 1.01) | 0.99 (0.99 - 1.00) |
|  | Higher | 1.03 (0.90 - 1.20) | **0.70 (0.55 - 0.90)** | **0.95 (0.91 – 1.00)** | 0.99 (0.97 – 1.00) | **0.99 (0.98 – 1.00)** | **0.99 (0.97 – 1.00)** |
| **Current student** | | | | | | | |
|  | No (referent) |  |  |  |  |  |  |
|  | Yes | 1.04 (0.95 - 0.14) | **0.77 (0.67 - 0.88)** | **0.97 (0.93 – 1.00)** | 1.00 (0.98 - 1.00) | **1.00 (0.99 - 1.00)** | 0.99 (0.98 – 1.00) |

Notes: results from multilevel logistic regression models. Results are presented as adjusted prevalence ratios (PR) and 95% confidence intervals (CI). Significant results (p<0.05) are presented in bold.
